# The Possible Carcinogenic Effects of Radiations on the Uterus

**DOI:** 10.1038/bjc.1970.91

**Published:** 1970-12

**Authors:** C. C. Bird, R. A. Willis

## Abstract

**Images:**


### THE POSSIBLE CARCINOGENIC EFFECTS OF RADIATIONS ON THE UTERUS

C. C. BIRD AND R. A. WILLIS

*From the Department of Pathology, University Medical Buildings, Foresterhill, Aberdeen, and the Department of Pathology, Imperial Cancer Research Fund, Lincoln's Inn Fields, London, W.C.2*

Received for publication July 21, 1970

**SUMMARY.—The carcinogenic effects of radium and X-rays on the rat uterus have been investigated. Malignant endometrial tumours, usually adeno-carcinomas, were produced in a small proportion of treated rats. One rat treated with X-rays developed an adeno-sarcoma (possibly carcino-sarcoma) of the endometrium. Benign mixed polypoidal endometrial tumours occurred also in radium and X-ray treated rats and in non-radiated controls; radiation increased the incidence of these tumours and may have induced malignant transformation in some. The incidence of lymphosarcomas and mammary tumours in the strain of rat used appeared to be influenced by radiation treatment.**

**Review of the literature of human cases of mixed uterine tumours showed that in women over 40 years, more than one-fifth of the reported cases had a history of previous pelvic radiation; with other kinds of uterine malignancy a history of prior radiation treatment was considerably less. The results of our experiments enhance the suspicion that radiations are one factor in the causation of uterine cancer, especially mixed tumours.**

MANY cases have now been reported of mixed uterine tumours (carcino-sarcomas and mixed mesodermal sarcomas) in women with a history of treatment some years previously with intra-uterine radium or abdominal X-irradiation for menopausal disturbances or other benign conditions (Sophian, 1932; Speert and Peightal, 1949; Hill and Miller, 1951; McElin and Davis, 1952; Kight, 1953; Klein, 1953; Symmonds and Dockerty, 1955; Taylor, 1958; Wolfe and Pedowitz, 1958; Krupp *et al.*, 1961; Boutselis and Ullery, 1962; Edwards *et al.*, 1963; Norris and Taylor, 1965; Rachmaninoff and Climie, 1966; Bartsich, O'Leary and Moore, 1967; Masterson and Kremper, 1969; Thomas, Harris and Enden, 1969; Schaepman-van Geuns, 1970). A survey of the literature revealed that well over one hundred such cases have been reported in the English language to date.

Other workers have studied the subsequent incidence of uterine cancer over a number of years in groups of women treated with radium of X-rays for benign conditions of the uterus. Some of these studies, to be cited later, have suggested an increased incidence, while others have not.

This report describes an experimental investigation of the carcinogenic effects of radium and X-rays on the rat uterus, with the special object of determining whether or not mixed endometrial tumours can be induced by radiations.

### MATERIALS AND METHODS

Female hooded rats, 2–5 months of age, developed from crosses between a white Wistar and a wild brown rat were used in all experiments. Experimental rats and controls were matched for age and weight as far as possible. They were fed MRC diet 41-B and given water *ad libitum.*

The rats were divided into three groups according to treatment: (a) intra-uterine radium, (b) direct X-irradiation of the uterus, and (c) X-irradiation of terine allografts inserted in the lower abdominal wall.

In the rats which received radium treatment anaesthesia was induced with a mixture of chloroform and ether (equal parts); a 2 mg. radium needle (4·2 cm. total length and 3·0 cm. active length) was inserted *per vaginam* into one uterine horn and retained by packing the vagina with gauze and suturing the labia. The dose delivered to the uterus calculated from the isodose curves for the radium needle was 960 rad. over 48 hours. An identical needle made of brass without radium was used for controls.

The rats receiving direct X-irradiation of the uterus were anaesthetized with a freshly prepared 2·5% solution of Avertin (Winthrop Laboratories) given intra-peritoneally at a dose of 0·5 ml. per 100 g. body weight. The skin and rectus muscle of the lower part of the abdomen were incised in the midline; the left horn of the uterus and the ovary were mobilized and pulled through the abdominal wound and placed on a sterile gauze swab soaked in N-saline. The two halves of a lead shield, 6 cm. in diameter and 0·6 cm. in thickness, which had an aperture at the centre large enough to accommodate the uterine horn, were placed beneath the uterus to shield the remainder of the abdomen. Doses of 500 or 1000 rad. of X-rays were delivered to the exposed uterine horn, using a Kx-10 X-ray machine operating at 100 kV, 5 mA and a 1 mm. aluminium filter; the half-value layer of the X-ray beam was 2·2 mm. aluminium. Controls were treated in a similar way except that they were not given X-irradiation.

The allografts were segments of the whole uterine horn 2–3 mm. in length from healthy young adult hooded donors approximately 3 months old. Two allografts were inserted subcutaneously and two intramuscularly in the lower part of the abdominal wall near the midline using the technique described previously (Bird, 1970). In pilot experiments it was found that nearly two-thirds of such allografts survived for over 2 months and that no tumours developed in any allografts which were allowed to survive for periods up to 12 months (unpublished observations). Two weeks after grafting, the animals were examined and if palpable grafts were present, anaesthesia was induced with Avertin and the grafts were exposed to 500 or 1000 rad. of X-rays through the abdominal skin as described above, except that the remainder of the abdomen was not shielded and the abdominal viscera received a dose depending on their distance from the X-ray source The controls were treated in a similar fashion except that they did not have allografts inserted and they were not X-irradiated.

All of the rats were examined at intervals of 1–2 weeks and if they developed overt abdominal tumours or if they became obviously sick and seemed unlikely to survive they were killed. Otherwise all rats were allowed to survive until natural death when a complete necropsy was performed. All tumours found were examined histologically, and in the case of uterine tumours all of the tumour was blocked and sectioned at multiple levels.

### RESULTS

Only rats surviving for more than 26 weeks after treatment have been included in the results. The number of rats which developed tumours of any kind—uterine or other—in each group and the total number of tumours which were found are shown in Table I. It can be seen that rats treated with radium or

TABLE I.—*Tumours of all Kinds—Uterine and Others—After Treatment with Radium or X-rays*

| Treatment | No. rats treated | No. rats with tumours | Total No. of tumours |
|---|---|---|---|
| Radium | 20 | 11 | 15 |
| Controls | 10 | 4 | 5 |
| X-rays (500 rad.) | 10 | 5 | 5 |
| X-rays (1000 rad.) | 10 | 6 | 7 |
| Controls | 10 | 2 | 2 |
| Allografts: X-rays (500 rad.) | 19 | 15 | 25 |
| Allografts: X-rays (1000 rad.) | 19 | 15 | 29 |
| Controls | 20 | 11 | 14 |

X-rays developed tumours more frequently than those which had no radiation treatment. The various types of tumour and the number of rats in which these were found are shown in Tables II and III.

### *Uterine tumours*

As shown in Table II endometrial carcinomas were produced in a small proportion of the rats treated with radium (3/20), or after direct exposure of one uterine horn to a 1000 rad. dose of X-rays (1/10), or following a 500 rad. dose of X-rays to the lower abdomen (2/19). Grossly, the tumours produced diffuse thickening of the wall of the affected horn, usually with pyometra. Histologically all the tumours but one consisted of moderately well-differentiated adenocarcinomas of the endometrium (Fig. 1); the exception was an anaplastic squamous carcinoma in a rat treated with radium. With one exception the tumours widely infiltrated

TABLE II.—*Uterine Tumours Produced by Treatment with Radium or X-rays in the Rat*

| Treatment | No. rats treated | Type of tumour | No. rats with tumour |
|---|---|---|---|
| Radium | 20 | Adenocarcinoma | 2 |
| | | Squamous carcinoma | 1 |
| | | Endometrial polyp | 2 |
| Controls | 10 | — | 0 |
| X-rays (500 rad.) | 10 | Endometrial polyp | 1 |
| | | Leiomyoma | 1 |
| X-rays (1000 rad.) | 10 | Adenocarcinoma | 1 |
| | | Adeno-sarcoma | 1 |
| | | Endometrial polyp | 3 |
| Controls | 10 | — | 0 |
| Allografts: X-rays (500 rad.) | 19* | Adenocarcinoma | 2 |
| | | Endometrial polyp | 6† |
| | | Fibroma of cervix | 1 |
| Allografts: X-rays (1000 rad.) | 19* | Endometrial polyp | 6 |
| | | Leiomyoma | 1 |
| Controls | 20 | Endometrial polyp | 3† |

* No tumours developed in any of the uterine allografts.
† One rat had 2 endometrial polyps.

TABLE III.—*Extra-uterine Tumours found after Treatment with Radium or X-rays in the Rat*

| Treatment | No. rats treated | Type of tumour | No. rats with tumour |
|---|---|---|---|
| Radium | 20 | Lymphosarcoma | 3 |
| | | Mammary—adenocarcinoma | 1 |
| | | fibro-adenoma | 1 |
| | | fibroma | 1 |
| | | Thyroid adenoma | 2 |
| | | Squamous carcinoma nares | 2 |
| Controls | 10 | Lymphosarcoma | 3 |
| | | Thyroid adenoma | 2 |
| X-rays (500 rad.) | 10 | Lymphosarcoma | 3 |
| X-rays (1000 rad.) | 10 | Lymphosarcoma | 1 |
| | | Renal carcinoma | 1 |
| Controls | 10 | Lymphosarcoma | 1 |
| | | Squamous carcinoma nares | 1 |
| Allografts: X-rays (500 rad.) | 19 | Lymphosarcoma | 6 |
| | | Mammary—adenocarcinoma | 2 |
| | | fibro-adenoma | 1* |
| | | Granulosa-cell tumour ovary | 1 |
| | | Fibrosarcoma skin | 1 |
| | | Squamous carcinoma nares | 1 |
| Allografts: X-rays (1000 rad.) | 19 | Lymphosarcoma | 7 |
| | | Mammary—adenocarcinoma | 3† |
| | | fibro-adenoma | 5 |
| | | Granulosa-cell tumour ovary | 1 |
| | | Luteoma ovary | 2 |
| | | Thyroid adenoma | 1 |
| | | Renal carcinoma | 1 |
| Controls | 20 | Lymphosarcoma | 6 |
| | | Mammary—fibro-adenoma | 2 |
| | | Fibrosarcoma skin | 1 |
| | | Thyroid adenoma | 1 |

* Four separate fibroadenomas were present.
† In 2 rats there were 2 widely separate adenocarcinomas.

the adjacent uterine tissues and produced widespread metastases in the peritoneum and abdominal lymph nodes. The exception was a polypoid adenocarcinoma of the endometrium which followed direct treatment of the uterus with a 1000 rad. dose of X-rays: in this case the structure of the tumour showed focal carcinomatous change at the tip of an endometrial polyp similar to those described below.

A sarcomatous tumour was also found in the group in which the uterus was directly exposed to a 1000 rad. dose of X-irradiation. In gross form this tumour was polypoidal and attached to the endometrial surface by a broad pedicle. Histologically, its general features were similar to those of the composite endometrial polyps, with both epithelial and non-epithelial components (Fig. 2). The epithelial component was glandular, partly cystic, and composed of columnar cells which in places showed atypical features with nuclear pleomorphism and numerous mitoses. The sarcomatous component was compact, highly vascular in places, and composed of plump polyhedral or spindle cells with many mitoses (Fig. 3). At the base of the pedicle the sarcomatous elements had penetrated the myometrium and infiltrated the adjacent mesometrium; no metastases were found. Contained within parts of the sarcomatous tissue there were irregular clumps of pleomorphic tumour cells surrounded by hyalinized collagenous tissue (Fig. 4). While it is possible that these were sarcomatous elements enclosed by collagenous stroma, it is also possible that they were malignant epithelial clumps which had evoked a stromal reaction. Serial sections of the tumour and special staining techniques failed to settle the question. Whatever the nature of these clumps, the tumour is regarded as a mixed one with an active epithelial component along with the sarcomatous one—at least an adeno-sarcoma. None of the rats in the control groups developed malignant uterine tumours.

As shown in Table II, endometrial polyps occurred in a porportion of the rats in all of the groups which received radium or X-ray treatment; in the non-radiated rats endometrial polyps of essentially similar structure were found only in the allograft control group. Grossly, the polyps measured 1·5–3 cm. in length and 0·5–2 cm. diameter at the tip; they consisted either of a solid lobulated pedunculated growth or of a cluster of smaller polyps attached to the endometrial surface by a broad pedicle. Histologically, they had a composite structure in which the epithelial elements formed a prominent part of the growth in nearly every case (Fig. 5). The epithelial tissues consisted of well-differentiated glandular structures, varying in size and frequently cystic; they were lined by cuboidal or columnar epithelium which was hyperplastic in places, and in approximately one-third of cases showed some squamous metaplasia. The stromal component was abundant in most cases and consisted of mature fibrous tissue along with myxomatous areas of more compact spindle or polyhedral cells especially around the glands (Fig. 6). Other differentiated elements found in about one-third of the polyps were adipose tissue (Fig. 7) and smooth muscle (Fig. 8); and in some cases the pedicle contained a prominent core of smooth muscle. Sections at multiple planes of these polyps showed no frankly malignant tissues; the appearances were essentially those of benign mixed endometrial tumours.

17/76 (22%) of the subcutaneous and 36/76 (47·4%) of the intramuscular allografts were viable histologically at the time of necropsy, although in most cases they were cystic and the endometrium atrophic; some of the grafts had survived for periods of over 2 years. However, no malignant tumours developed in any of the surviving allografts. Two rats in the X-irradiated groups were found to have uterine leiomyomata and one a cervical fibroma at necropsy (Table II).

*Other tumours*

A variety of other tumours occurred in both the irradiated and control rats (Table III). Lymphoblastic lymphosarcomas involving principally the mesenteric lymph nodes and less often other intra-abdominal and mediastinal lymph nodes and viscera, occurred with nearly equal frequency in treated rats and controls, 25·6% and 22·5% respectively. However, in the group treated by abdominal X-irradiation the mean tumour-induction time was significantly reduced: 44·6 weeks and 50 weeks with the 500 and 1000 rad. dose respectively, compared with 73·6 weeks in controls ($P < 0{\cdot}01$ and $< 0{\cdot}05$, respectively). Mammary tumours, principally adenocarcinomas and fibroadenomas, were found most frequently in the allografted X-irradiated rats (11/38). Most of the tumours arose from the mammary pads within the field of irradiation in the lower abdomen. Similar tumours were found in a few rats of the radium-treated group (3/20) and the allograft control group (2/20). Ovarian tumours of granulosa-cell type and luteomas were found in a few of the allografted X-irradiated rats (4/38). One of the granulosa-cell tumours showed, in addition to the typical granulosa-cell sheets, tubular and papillary carcinoma which had metastasized to the peritoneum and abdominal lymph nodes. The endometrium in all of the rats with ovarian tumours was hyperplastic; but we cannot be sure that this resulted from ovarian hormone hypersecretion, because hyperplastic changes are frequent in the endometrium of old rats and were found in nearly 40% of the control rats in this series.

Various other tumours were found sporadically both in the groups receiving radiation, and in non-radiated controls (Table III) and these had no apparent association with treatment.

### DISCUSSION

Spontaneous tumours of the uterus occur infrequently in the rat; adenocarcinomas, squamous carcinomas, myosarcomas, fibrosarcomas, carcino-sarcomas, myomas and fibromas have all been recorded, the incidence varying with the strain of rat studied (Bullock and Curtis, 1930; Curtis, Bullock and Dunning, 1931; Ratcliffe, 1940; Crain, 1958; Gilbert and Gillman, 1958; Thompson and Hunt, 1963; Franks, 1967). Endometrial polyps, on the other hand, have been reported as of frequent occurrence in some strains of rat (Snell, 1965; Gellatly, 1967); these are usually small polypoidal growths and have been classed as adenomatous polyps or fibro-adenomas of the endometrium.

Various chemical agents have been used to study the induction of epithelial and non-epithelial tumours in the rat uterus (Vellios and Griffin, 1957; Mori, 1964; Shintani, Glass and Page, 1966; Baba and Von Haam, 1967; Castro, Fechner and Spjut, 1968; Alexandrov, 1969). However, the possibility of radiations having a carcinogenic effect on the uterus has rarely been studied experimentally. Lorenz *et al.* (1947) and Lorenz (1950) reported the induction of uterine carcinomas in rabbits by $\gamma$-rays from an external radium source. Uterine carcinomas and sarcomas after exposure to whole body X-irradiation were reported in mice by Deringer, Lorenz and Uphoff (1955), and in rats by Binhammer *et al.* (1957). There have been no previous studies on tumour induction by direct exposure of the uterus to radiations.

Our experiments show that in the rat malignant uterine tumours, usually endometrial adenocarcinomas, can be induced by intra-uterine radium application or by X-irradiation of the uterus from without. Furthermore, in one rat where

---

### EXPLANATION OF PLATES

FIG. 1.—Typical endometrial adenocarcinoma produced by radium or X-ray treatment. × 335.

FIG. 2.—Adeno-sarcoma of endometrium produced by exposure of uterus to 1,000 rad. dose of X-rays. Glands lined by columnar epithelium with pleomorphic nuclei, along with spindle-cell sarcomatous tissue. × 335.

FIG. 3.—Adeno-sarcoma of endometrium. Sarcomatous tissue composed of polyhedral and spindle cells showing many mitoses. × 335.

FIG. 4.—Adeno-sarcoma of endometrium. Irregular clumps of pleomorphic tumour cells enclosed by hyalinized collagenous stroma (right). × 335.

FIG. 5.—Composite endometrial polypus composed of glandular elements, some of which are cystic, and fibrous tissue. × 85.

FIG. 6.—Endometrial polypus. Myxomatous tissue (right) and more compact spindle- or polyhedral-cell tissue surrounding the glands. × 335.

FIG. 7.—Endometrial polypus. Adipose elements in the fibrous tissue. × 335.

FIG. 8.—Endometrial polypus. Fasciculated spindle-cell tissue with tinctorial properties of smooth muscle. × 335.

1

2

3

4

Bird and Willis.

5

6

Bird and Willis.

7

8

the uterus was exposed to direct X-irradiation, a composite endometrial tumour classed as an adeno-sarcoma was produced. Whilst this tumour was not structurally identical with the mixed endometrial tumours of women, its composite structure and the possibility that it may also have contained carcinomatous areas, suggests that it may well represent the rat counterpart of the human neoplasm.

The failure to induce any tumours in the uterine allografts is surprising especially since adenocarcinomas were induced in the uterus within the abdomen in a few of the allografted rats. Unless allografted tissues react differently to carcinogenic stimuli there is no satisfactory explanation for this finding.

As in the human subject (Willis, 1967), the great size and composite structure of the large localized endometrial polyps in rats suggest that they are well-differentiated benign mixed tumours. Since these tumours were found both in the rats treated with radiations and in non-radiated controls, perhaps these benign polyps represent the usual mixed tumour of the rat endometrium, the spontaneous incidence of which can be increased by exposure to radiations—which also, though less commonly, induce carcinomatous or sarcomatous change, or rarely both.

It seems probable also that in these experiments radiation treatment promoted the induction of lymphosarcomas and mammary tumours, especially in the groups exposed to abdominal X-irradiation. The total incidence of lymphosarcoma was not significantly increased but the mean induction time was reduced significantly. The induction of lymphosarcomas in mice by radiations is well-established (Kaplan, 1948). The incidence of mammary tumours was greater in rats treated by abdominal X-irradiation than in controls and the mean induction time of tumours was less in the irradiated group than in controls; but the number of tumour-bearing animals was too small for statistical analysis. Radiations have been shown, however, to induce mammary tumours in rats (Bond *et al.*, 1960; Telles and Ward, 1969). Granulosa-cell tumours and luteomas of ovary also occurred in the group receiving abdominal X-irradiation. From this small series the role of X-irradiation in their induction cannot be positively asserted; but the carcinogenic effects of X-rays on ovarian tissues in mice is well established (Furth and Boon, 1947; Furth and Sobel, 1947; Deringer, Lorenz and Uphoff, 1955).

The role of pelvic radiation in the induction of human uterine malignancy has still to be established. The most suggestive association exists between radiation treatment of the uterus for benign menstrual disturbances and the development some years later of a mixed endometrial tumour.

On reviewing the literature it was found that, if mixed tumours of the uterus of children and young women under 40 were excluded, approximately 750 cases of mixed tumours (carcino-sarcomas and mixed mesodermal sarcomas) of the uterine corpus and cervix have been reported in the English language to date. Where it was possible the reports of all these cases were reviewed to establish whether or not there was a past history of pelvic radiation: 141 (22%) of 640 cases where positive information was given as to whether or not there had been previous pelvic radiation, had received such treatment. The mean latent interval between radiation treatment and the diagnosis of the tumour was 11·6 years, and at the time of diagnosis the mean age of cases with previous pelvic radiation was 55·3 years compared with 60·8 years in those without. By comparison, the published data of other types of uterine malignancy, where the possible carcinogenic effects of radiations have been noted, show that the incidence of previous pelvic radiation was 8% in nearly 300 cases of leiomyosarcoma and 4% in approximately 4500 of endometrial carcinoma. Thus mixed tumours of the uterus, mainly endometrial in origin, have a history of previous pelvic radiation in a much higher proportion of cases than do other forms of uterine malignancy; and this, together with the fact that the mean age of cases with previous pelvic radiation is somewhat lower than those without, strongly suggests that pelvic radiation plays a significant role in the induction of some mixed uterine tumours—though clearly not in all. (We ourselves have studied 23 cases of mixed endometrial tumours, in 16 of these it was possible to determine whether or not there had been previous radiation and in four cases such treatment had been given. We hope to publish these later, along with a review of the subject.)

Other workers have studied the long-term incidence of uterine malignancy in groups of women who had received treatment with radium or X-rays for benign uterine conditions, and have compared this with the spontaneous incidence in the general population. Many such studies have been invalidated, however, by the failure to follow patients for a sufficiently long period after treatment, since the latent interval between exposure to radiation and the development of a tumour may be many years. Difficulties have also been encountered in obtaining strictly comparable groups of the general population for comparison. Despite these difficulties, some workers have suggested that the incidence of uterine carcinoma may be increased after pelvic radiation (Corscaden, Fertig and Gusberg, 1946; Palmer and Spratt, 1956; Copeland, Nelson and Payne, 1957). Others, have been unable to convince themselves of such an association (Smith and Bowden, 1948; Hunter *et al.*, 1954; Turnbull, 1956); while others, although they have noted a slight increase in the uterine cancer rate (Stander, 1957; Rubin, Ryplansky and Dutton, 1961; Paloucek *et al.*, 1963; Doll and Smith, 1968) have either considered this of doubtful significance or have related it to the predisposing abnormality of the uterus which necessitated the original radiation treatment. However, the strong suspicion remains that pelvic radiation may result in an increased long-term incidence of uterine cancer, especially of mixed tumours, and the results of our experiments strengthen this suspicion.

We are grateful to Professors T. Symington and A. R. Currie for facilities to carry out this work in the Pathology Departments at the Glasgow Royal Infirmary and the University of Aberdeen. We wish to thank Dr J. Glennie, Glasgow Royal Infirmary Department of Radiotherapy, for advice and arranging facilities to carry out the radiation of animals. Our sincere thanks are due also to Miss A. M. Craise, Superintendent Radiographer, Glasgow Royal Infirmary Department of Radiotherapy, for assistance with the X-irradiation of the animals. This work was supported by a grant from the Scottish Hospital Endowments Research Trust.

### REFERENCES

ALEXANDROV, V. A.—(1969) *Nature, Lond.*, **222**, 1064.
BABA, N. AND VON HAAM, E.—(1967) *Prog. exp. Tumor Res.*, **9**, 192.
BARTSICH, E. G., O'LEARY, J. A. AND MOORE, J. G.—(1967) *Obstet. Gynec., N.Y.*, **30**, 518.
BINHAMMER, R. T., FINERTY, J. C., SCHNEIDER, M. AND CUNNINGHAM, A. W.—(1957) *Radiat. Res.*, **6**, 339.
BIRD, C. C.—(1970) *J. Path.*, **100**, 105.

BOND, V. P., SHELLABARGER, C. J., CRONKITE, E. P. AND FLIEDNER, T. M.—(1960) *Radiat. Res.*, **13**, 318.
BOUTSELIS, J. G. AND ULLERY, J. C.—(1962) *Obstet. Gynec., N.Y.*, **20**, 23.
BULLOCK, F. D. AND CURTIS, M. R.—(1930) *J. Cancer Res.*, **14**, 1.
CASTRO, H. F., FECHNER, R. E. AND SPJUT, H. J.—(1968) *Archs Path.*, **86**, 475.
COPELAND, W. E., NELSON, P. K. AND PAYNE, F. L.—(1957) *Am. J. Obstet. Gynec.*, **73**, 615.
CORSCADEN, J. A., FERTIG, J. W. AND GUSBERG, S. B.—(1946) *Am. J. Obstet. Gynec.*, **51**, 1.
CRAIN, R. C.—(1958) *Am. J. Path.*, **34**, 311.
CURTIS, M. R., BULLOCK, F. D. AND DUNNING, W. F.—(1931) *Am. J. Cancer*, **15**, 67.
DERINGER, M. K., LORENZ, E. AND UPHOFF, D. E.—(1955) *J. natn. Cancer Inst.*, **15**, 931.
DOLL, R. AND SMITH, P. G.—(1968) *Br. J. Radiol.*, **41**, 362.
EDWARDS, D. L., STERLING, L. N., KELLER, R. H. AND NOLAN, J. F.—(1963) *Am. J. Obstet. Gynec.*, **85**, 1002.
FRANKS, L. M.—(1967) In ' Pathology of Laboratory Rats and Mice '. Edited by E. Cotchin and F. J. C. Roe. Oxford and Edinburgh (Blackwell Scientific Publications), p. 469.
FURTH, J. AND BOON, M. C.—(1947) *Cancer Res.*, **7**, 241.
FURTH, J. AND SOBEL, H.—(1947) *Cancer Res.*, **7**, 246.
GELLATLY, J. B. M.—(1967) In ' Pathology of Laboratory Rats and Mice '. Edited by E. Cotchin and F. J. C. Roe. Oxford and Edinburgh (Blackwell Scientific Publications), p. 498.
GILBERT, C. AND GILLMAN, J.—(1958) *S. Afr. J. med. Sci.*, **23**, 257.
HILL, R. P. AND MILLER, F. N.—(1951) *Cancer, N.Y.*, **4**, 803.
HUNTER, R. M., LUDWICK, N. V., MOTLEY, J. F. AND OAKS, W. W.—(1954) *Am. J. Obstet. Gynec.*, **67**, 121.
KAPLAN, H. S.—(1948) *J. natn. Cancer Inst.*, **8**, 191.
KIGHT, J. R.—(1953) *Am. J. Obstet. Gynec.*, **66**, 733.
KLEIN, J.—(1953) *Am. J. Obstet. Gynec.*, **65**, 1212.
KRUPP, P. J., STERNBERG, W. H., CLARK, W. H., ST. ROMAIN, M. J. AND SMITH, R. C.—(1961) *Am. J. Obstet. Gynec.*, **81**, 959.
LORENZ, E., ESCHENBRENNER, A. B., HESTON, W. E. AND DERINGER, M. K.—(1947) Proc. Fourth *Int. Cancer Res. Congr.*, St. Louis, p. 117.
LORENZ, E.—(1950) *Am. J. Roentg.*, **63**, 176.
MCELIN, T. W. AND DAVIS, H.—(1952) *Am. J. Obstet. Gynec.*, **63**, 605.
MASTERSON, J. G. AND KREMPER, J.—(1969) *Am. J. Obstet. Gynec.*, **104**, 693.
MORI, K.—(1964) *Gann.*, **55**, 277.
NORRIS, H. J. AND TAYLOR, H. B.—(1965) *Obstet. Gynec., N.Y.*, **26**, 689.
PALMER, J. P. AND SPRATT, D. W.—(1956) *Am. J. Obstet. Gynec.*, **72**, 497.
PALOUCEK, F. P., RANDALL, C. L., GRAHAM, J. B. AND GRAHAM, S.—(1963) *Obstet. Gynec., N.Y.*, **21**, 530.
RACHMANINOFF, N. AND CLIMIE, A. R.—(1966) *Cancer, N.Y.*, **19**, 1705.
RATCLIFFE, H. L.—(1940) *Am. J. Path.*, **16**, 237.
RUBIN, P., RYPLANSKY, A. AND DUTTON, A.—(1961) *Am. J. Roentg.*, **85**, 503.
SCHAEPMAN-VAN GEUNS, E. J.—(1970) *Cancer, N.Y.*, **25**, 72.
SHINTANI, S., GLASS, L. E. AND PAGE, E. W.—(1966) *Am. J. Obstet. Gynec.*, **95**, 550.
SMITH, F. R. AND BOWDEN, L.—(1948) *Am. J. Roentg.*, **59**, 796.
SNELL, K. C.—(1965) In ' The Pathology of Laboratory Animals '. Edited by W. E. Ribelin and J. R. McCoy. Springfield, Illinois (Charles C. Thomas), p. 270.
SOPHIAN, L.—(1932) *Am. J. Obstet. Gynec.*, **24**, 911.
SPEERT, H. AND PEIGHTAL, T. C.—(1949) *Am. J. Obstet. Gynec.*, **57**, 261.
STANDER, R. W.—(1957) *Obstet. Gynec., N.Y.*, **10**, 223.
SYMMONDS, R. E. AND DOCKERTY, M. B.—(1955) *Surgery Gynec. Obstet.*, **100**, 232, 322.

TAYLOR, C. W.—(1958) *J. Obstet. Gynaec. Br. Commonw.*, **65**, 177.
TELLES, N. C. AND WARD, B. C.—(1969) *Radiat. Res.*, **37**, 577.
THOMAS, W. O., HARRIS, H. H. AND ENDEN, J. A.—(1969) *Am. J. Obstet. Gynec.*, **104**, 209.
THOMPSON, S. W. AND HUNT, R. D.—(1963) *Ann. N.Y. Acad. Sci.*, **108**, 833.
TURNBULL, A. C.—(1956) *J. Obstet. Gynaec. Br. Commonw.*, **63**, 179.
VELLIOS, F. AND GRIFFIN, J.—(1957) *Cancer Res.*, **17**, 364.
WILLIS, R. A.—(1967) ' Pathology of Tumours ', London (Butterworth & Co.) p. 555.
WOLFE, S. A. AND PEDOWITZ, P.—(1958) *Obstet. Gynec., N.Y.*, **12**, 54.


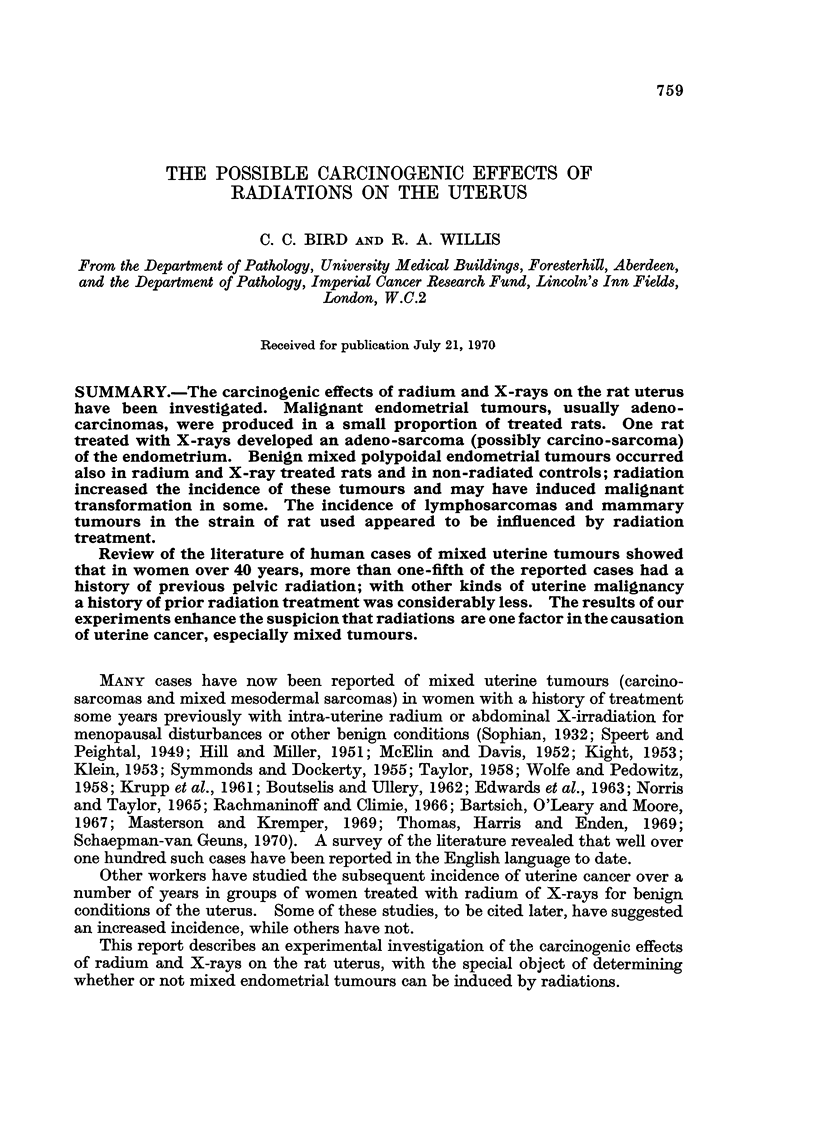

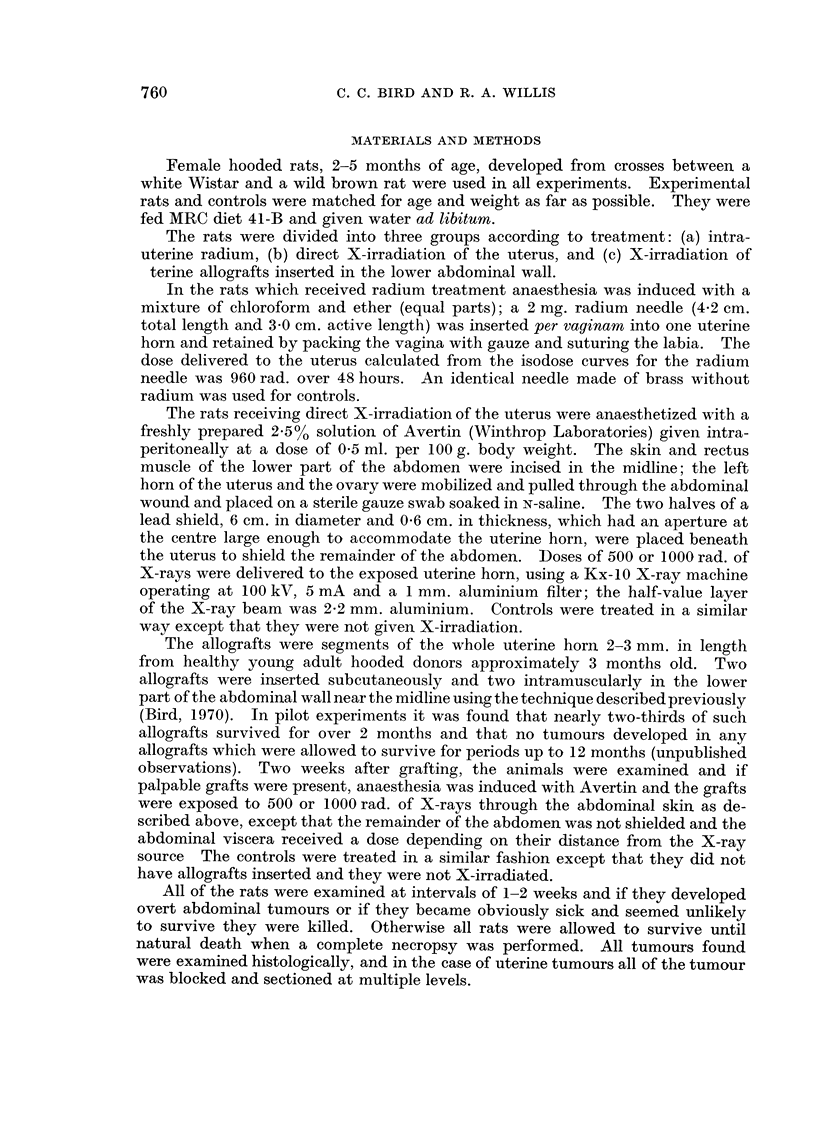

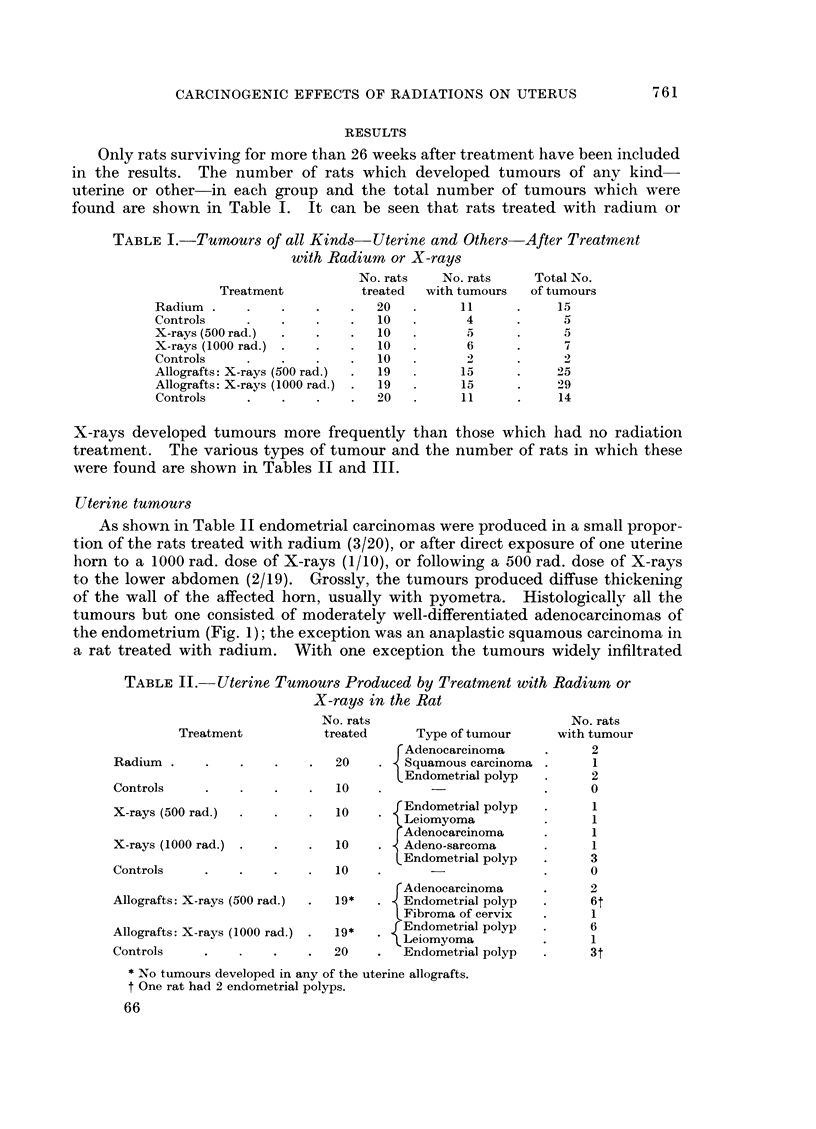

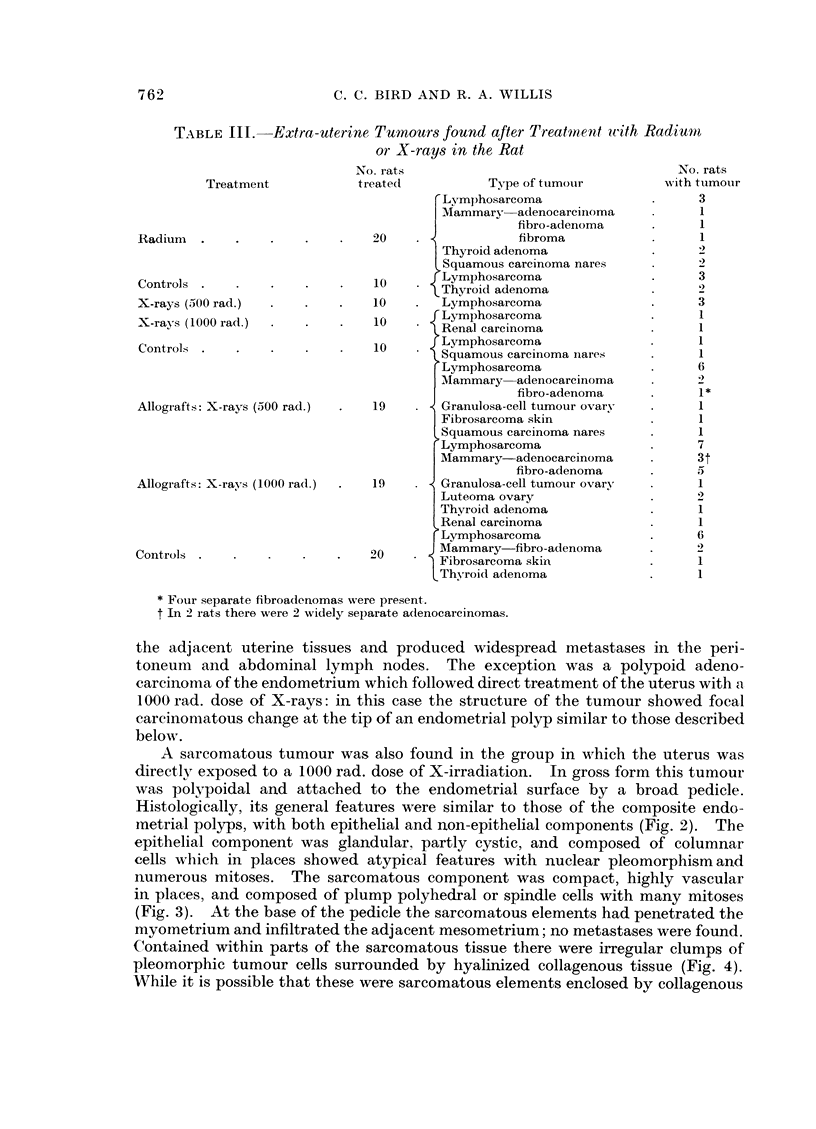

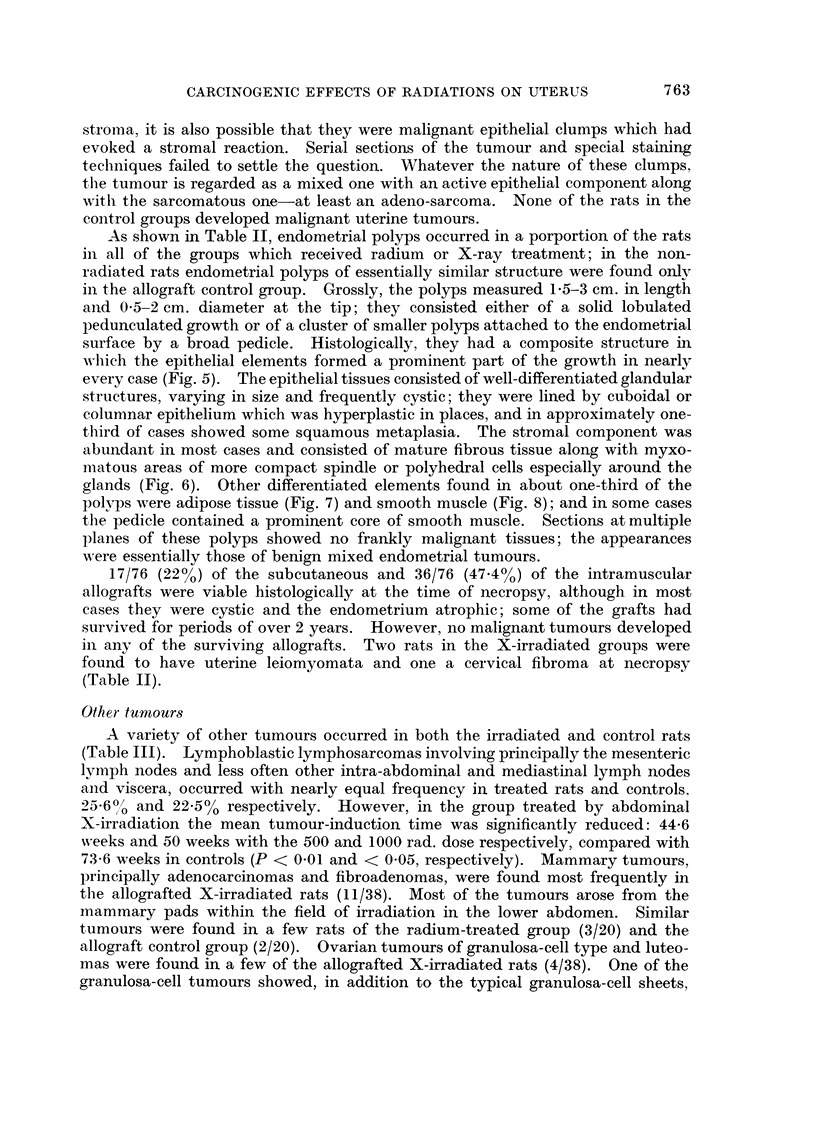

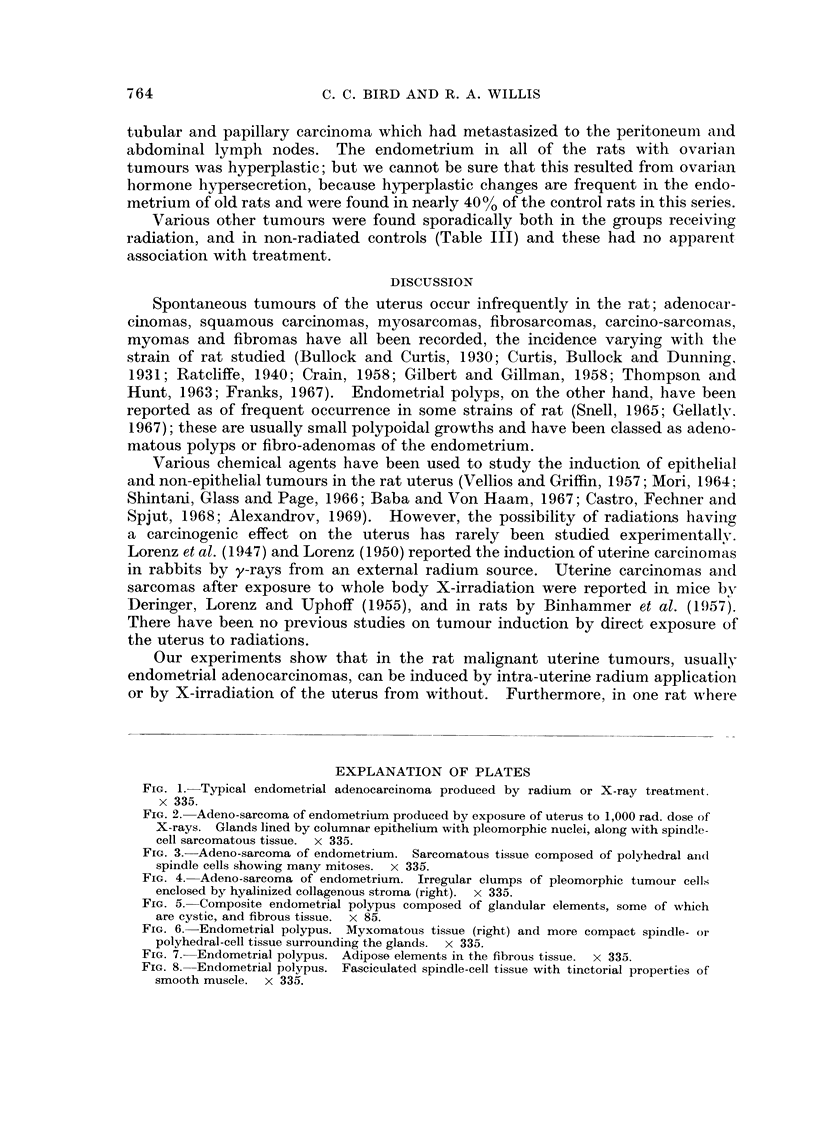

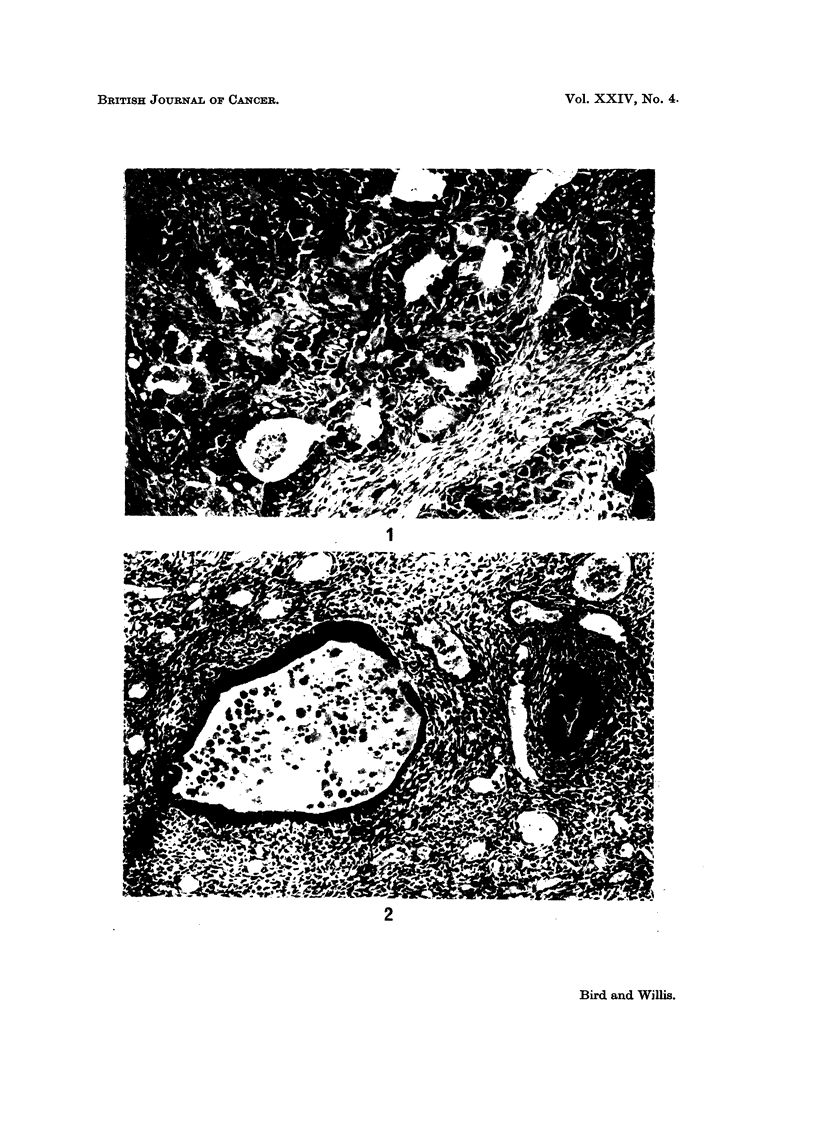

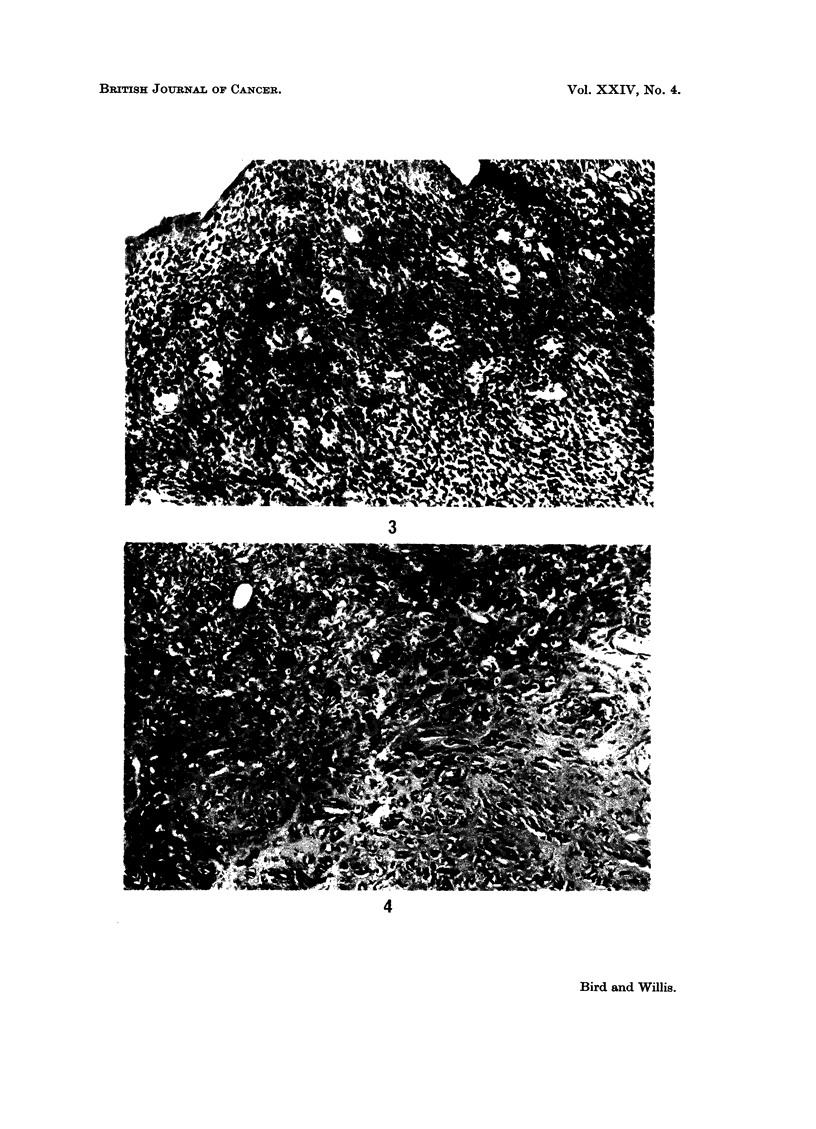

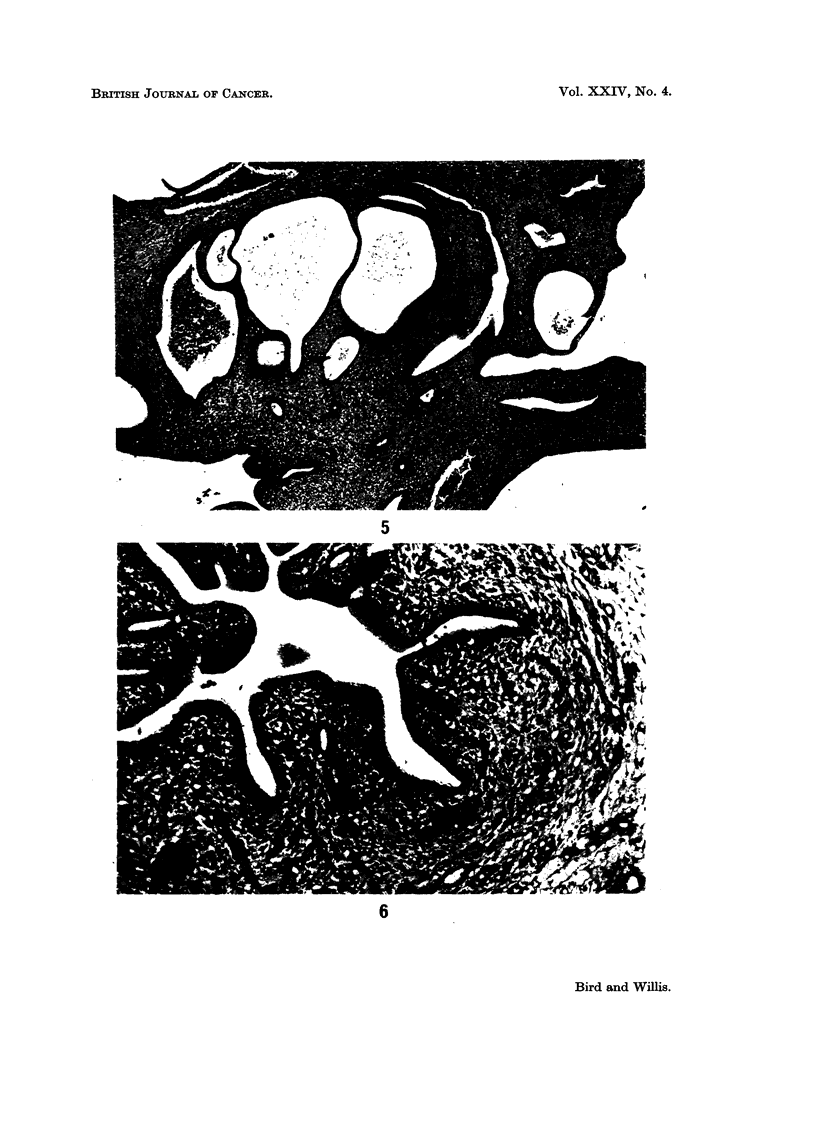

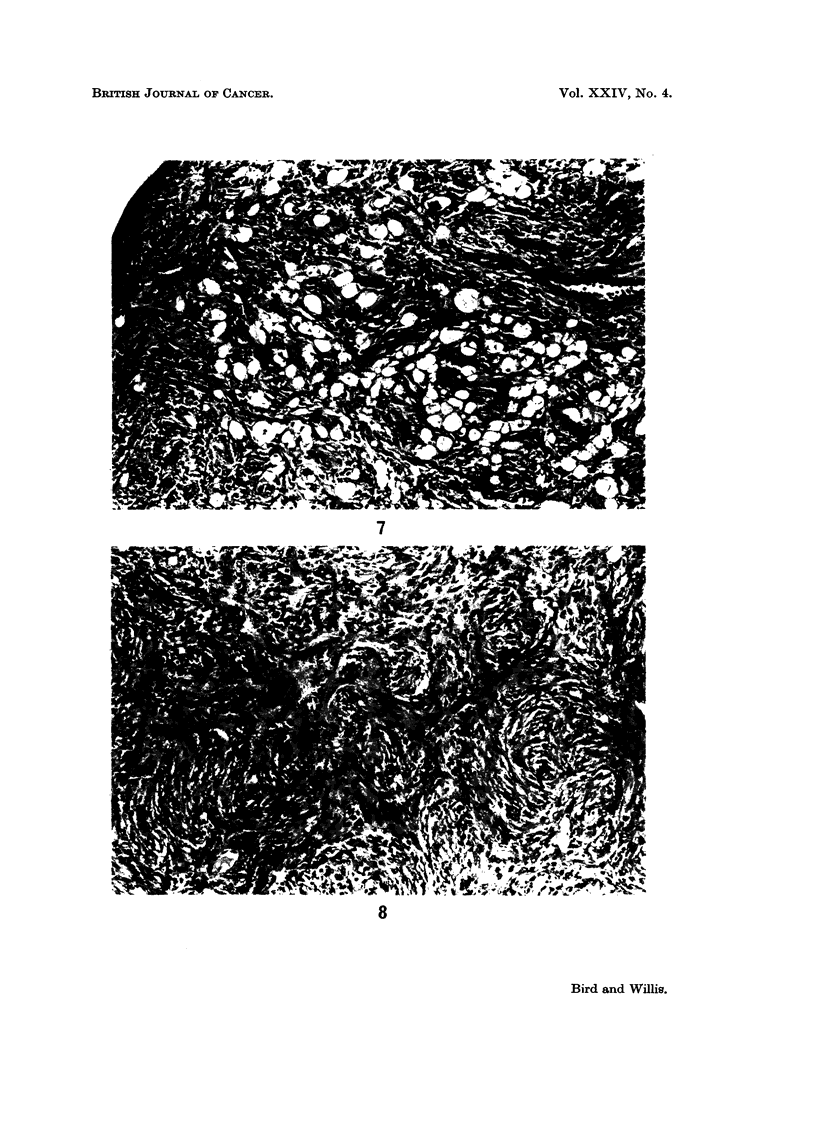

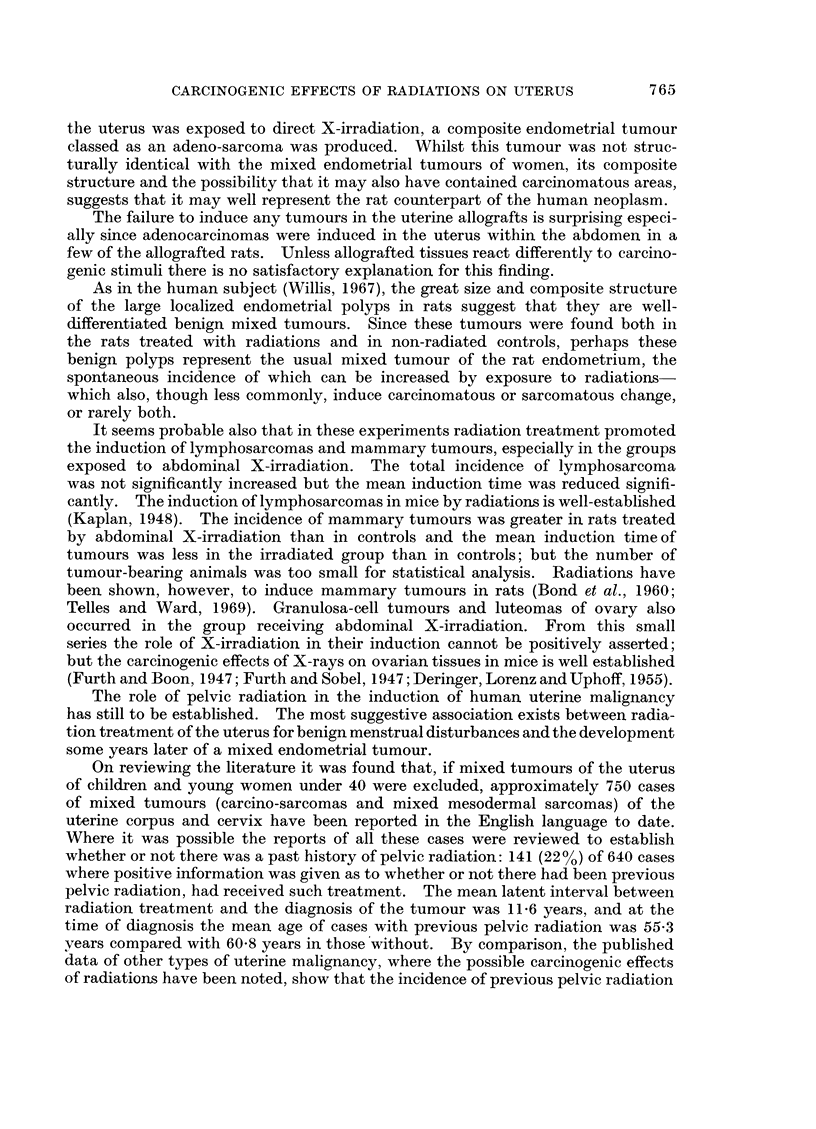

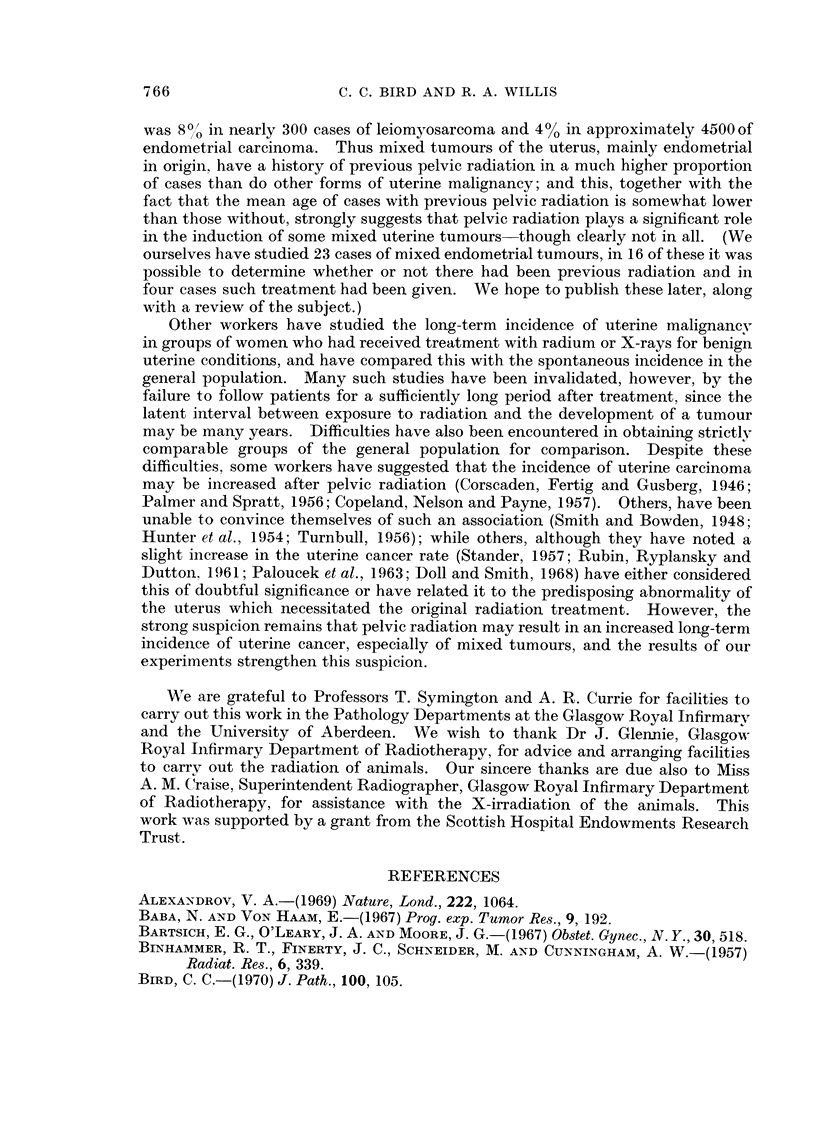

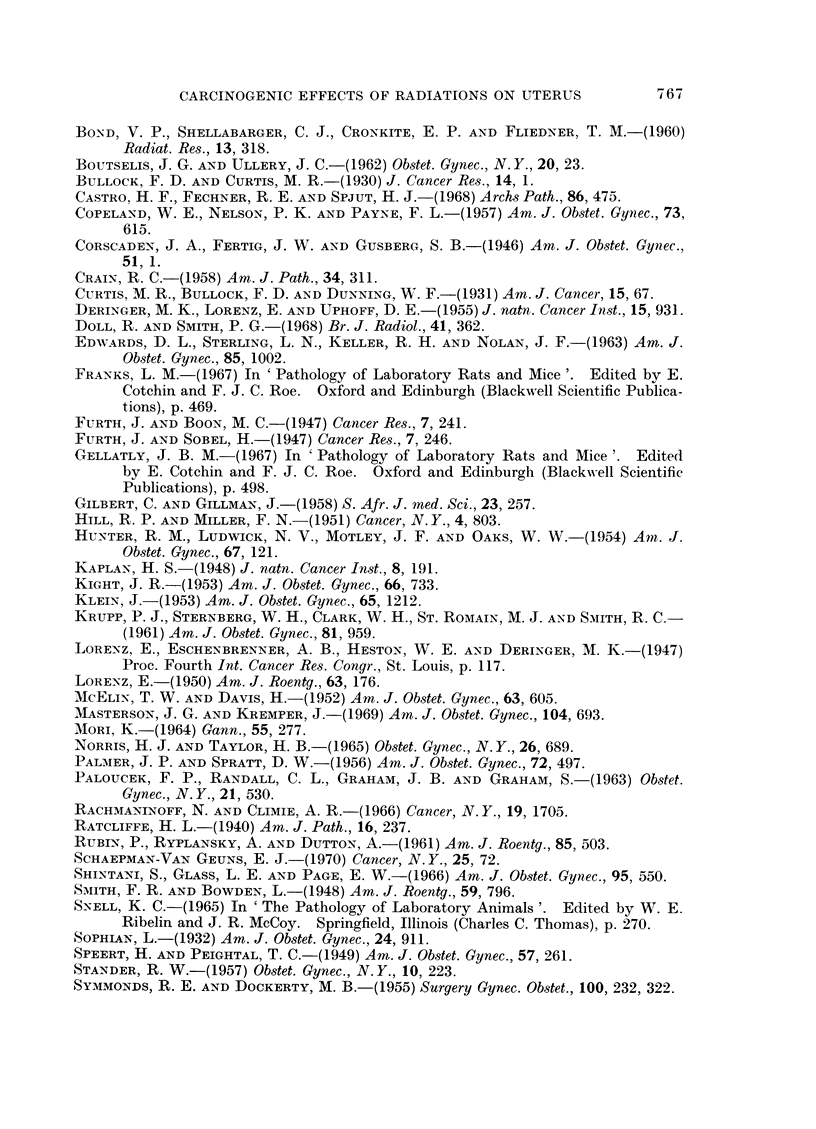

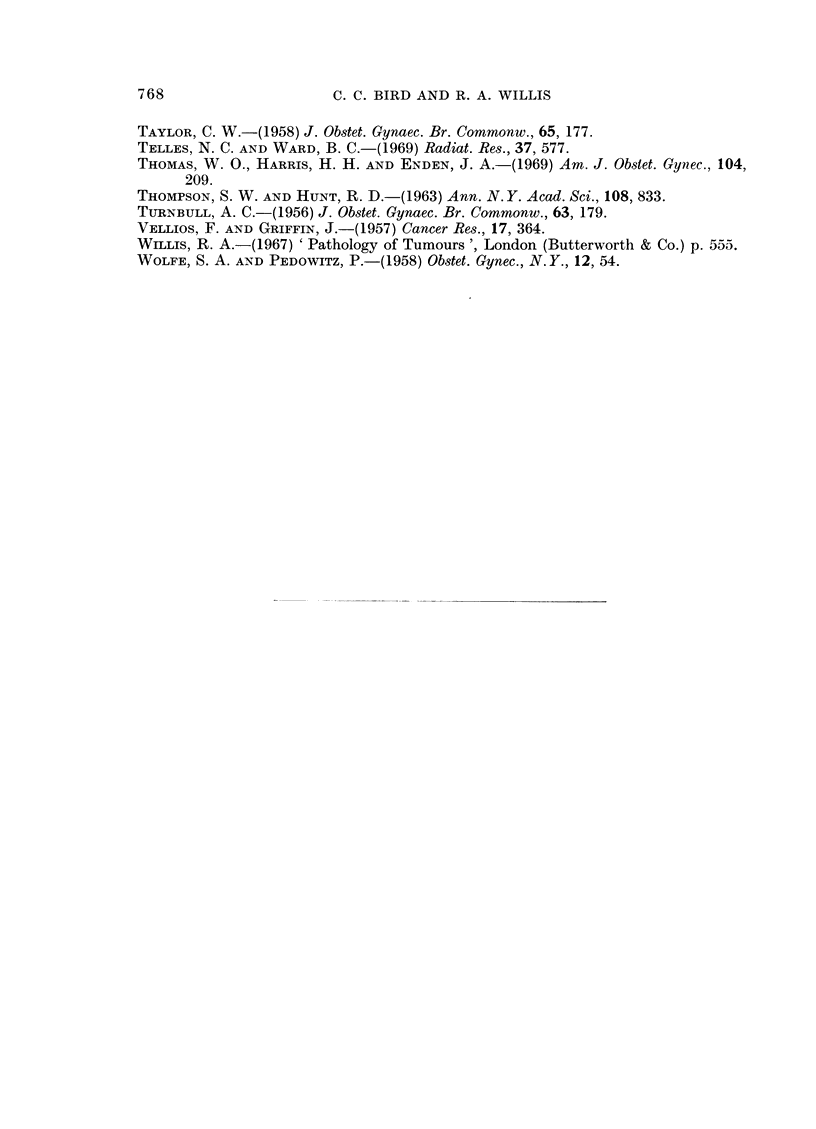


## References

[OCR_00618] BINHAMMER R. T., FINERTY J. C., SCHNEIDER M., CUNNINGHAM A. W. (1957). Tumor induction in rats by single total-body x-irradiation.. Radiat Res.

[OCR_00626] BOND V. P., SHELLABARGER C. J., CRONKITE E. P., FLIEDNER T. M. (1960). Studies on radiation-induced mammary gland neoplasia in the rat. 5. Induction by localized irradiation.. Radiat Res.

[OCR_00615] Bartsich E. G., O'Leary J. A., Moore J. G. (1967). Carcinosarcoma of the uterus. A 50-year review of 32 cases (1917-1966).. Obstet Gynecol.

[OCR_00622] Bird C. C. (1970). Histological studies of allografts of uterine tissues in rats.. J Pathol.

[OCR_00641] CRAIN R. C. (1958). Spontaneous tumors in the Rochester strain of the Wistar rat.. Am J Pathol.

[OCR_00633] Castro H. F., Fechner R. E., Spjut H. J. (1968). Induced mesenchymal tumors of the rat genital tract.. Arch Pathol.

[OCR_00646] Doll R., Smith P. G. (1968). The long-term effects of x irradiation in patients treated for metropathia haemorrhagica.. Br J Radiol.

[OCR_00666] HILL R. P., MILLER F. N. (1951). Combined mesenchymal sarcoma and carcinoma (carcinosarcoma) of the uterus.. Cancer.

[OCR_00670] HUNTER R. M., LUDWICK N. V., MOTLEY J. F., OAKS W. W. (1954). The use of radium in the treatment of benign lesions of the uterus; a critical twenty-year survey.. Am J Obstet Gynecol.

[OCR_00674] KLEIN J. (1953). Carcinosarcoma of the endometrium.. Am J Obstet Gynecol.

[OCR_00676] KRUPP P. J., STERNBERG W. H., CLARK W. H., ST ROMAN M. J., SMITH R. C. (1961). Malignant mixed Mullerian neoplasms (mixed mesodermal tumors).. Am J Obstet Gynecol.

[OCR_00688] MORI K. (1964). INDUCTION OF PULMONARY AND UTERINE TUMORS IN RATS BY SUBCUTANEOUS INJECTIONS OF 4-NITROQUINOLINE 1-OXIDE.. Gan.

[OCR_00685] McELIN T. W., DAVIS H. (1952). Mesodermal mixed tumor of the corpus uteri.. Am J Obstet Gynecol.

[OCR_00694] PALMER J. P., SPRATT D. W. (1956). Pelvic carcinoma following irradiation for benign gynecological diseases.. Am J Obstet Gynecol.

[OCR_00701] RUBIN P., RYPLANSKY A., DUTTON A. (1961). Incidence of pelvic malignancies following irradiation for benign gynecologic conditions.. Am J Roentgenol Radium Ther Nucl Med.

[OCR_00699] Rachmaninoff N., Climie A. R. (1966). Mixed mesodermal tumors of the uterus.. Cancer.

[OCR_00717] SYMMONDS R. E., DOCKERTY M. B. (1955). Sarcoma and sarcoma-like proliferations of the endometrial stoma. II. Carcinosarcoma.. Surg Gynecol Obstet.

[OCR_00705] Shintani S., Glass L. E., Page E. W. (1966). Studies of induced malignant tumors of placental and uterine origin in the rat. II. Induced tumors and their pathogenesis with special reference to choriocarcinoma.. Am J Obstet Gynecol.

[OCR_00726] THOMPSON S. W., HUNT R. D. (1963). SPONTANEOUS TUMORS IN THE SPRAGUE-DAWLEY RAT: INCIDENCE RATES OF SOME TYPES OF NEOPLASMS AS DETERMINED BY SERIAL SECTION VERSUS SINGLE SECTION TECHNICS.. Ann N Y Acad Sci.

[OCR_00727] TURNBULL A. C. (1956). Radiation menopause or hysterectomy. II. Mortality, reliability and subsequent pelvic cancer.. J Obstet Gynaecol Br Emp.

[OCR_00720] Telles N. C., Ward B. C. (1969). The effects of radiation and ethionine on rat mammary tumor incidence.. Radiat Res.

[OCR_00728] VELLIOS F., GRIFFIN J. (1957). The pathogenesis of dimethylbenzanthracene-induced carcinoma of the cervix of rats.. Cancer Res.

